# Habits of Cotton Worm

**Published:** 1875-04

**Authors:** 


Habits of the Cotton-Worm.—According to Mr. Aug. R. Grote, the cotton-worm dies out every year, with its food-plant, and its next appearance is always the result of immigration. He has observed that the appearance of the worm in the cotton-fields is always heralded by flights of the moth. The worm is always heard of to the southward at first, and never to the northward of any given locality in the cotton-belt. Mr. Grote never could discover any traces of the insect in any stage during the months *preceding* the appearance of the first brood heralded by the moth and *after* the cotton was above ground. Hence he concludes that while the cotton-plant is not indigenous to the Southern States (where it becomes an annual), the cotton-worm moth may be esteemed not a denizen but a visitant, brought by various causes to breed in a strange region, and that it naturally dies out in the cotton-belt, unable to suit itself *as yet* to the altered economy of its food-plant and to contend with the changes of our seasons. Possibly in the southern portions of Texas, or in the Floridian peninsula, the cotton-worm may be able to sustain itself during the entire year. Its true home, however, appears to be the West Indies, Mexico, and Brazil, where the cotton-plant is perennial. *Popular Science Monthly.*

Livers Mended.—An English physician, says the Danbury *News*, recently removed a section of a patient's liver, placed it on a plate, scraped it carefully and returned it to its place, fully restored to its normal action. This promises to work a revolution in the treatment of disease, and in a few years we will have an addition to domestic literature something like this: "Husband, I wish you would take John's right lung down to the dôctor's this morning, and have the middle valve fixed; or, "Will you stop into the doctor's when you come home this noon, and see if he has Mary's liver mended, as she wants to go out to tea this evening. The practice will become so common in time, we are sure, that none of the neighbors will be in any way startled to see a wife, with a veil tied around her head, leaning out of a bedroom window, and shouting to a receding husband: "Je-remiah! tell Dr. Scrapem to send up William's right kidney at once, whether it is done or not. He's had it there more'n a week, and the child might as well be without any kidney, and done with it!"

Shoe-leather, chemically considered, is oxide of beef.

